# Rationale for a New Generation of Indicators for Coastal Waters

**DOI:** 10.1289/ehp.6903

**Published:** 2004-05-11

**Authors:** Gerald Niemi, Denice Wardrop, Robert Brooks, Susan Anderson, Valerie Brady, Hans Paerl, Chet Rakocinski, Marius Brouwer, Barbara Levinson, Michael McDonald

**Affiliations:** ^1^Natural Resources Research Institute, University of Minnesota, Duluth, Minnesota, USA; ^2^Cooperative Wetlands Center, Pennsylvania State University, University Park, Pennsylvania, USA; ^3^Bodega Marine Laboratory, University of California Davis, Bodega Bay, California, USA; ^4^Institute of Marine Sciences, University of North Carolina-Chapel Hill, Morehead City, North Carolina, USA; ^5^Department of Coastal Sciences, University of Southern Mississippi, Ocean Springs, Mississippi, USA; ^6^U.S. Environmental Protection Agency, National Center for Environmental Research, Washington DC, USA; ^7^U.S. Environmental Protection Agency, Environmental Monitoring and Assessment Program, Research Triangle Park, North Carolina, USA

**Keywords:** coastal, ecologic, estuarine, health, indicators, marine, nutrients, responses, stressors

## Abstract

More than half the world’s human population lives within 100 km of the coast, and that number is expected to increase by 25% over the next two decades. Consequently, coastal ecosystems are at serious risk. Larger coastal populations and increasing development have led to increased loading of toxic substances, nutrients and pathogens with subsequent algal blooms, hypoxia, beach closures, and damage to coastal fisheries. Recent climate change has led to the rise in sea level with loss of coastal wetlands and saltwater intrusion into coastal aquifers. Coastal resources have traditionally been monitored on a stressor-by-stressor basis such as for nutrient loading or dissolved oxygen. To fully measure the complexities of coastal systems, we must develop a new set of ecologic indicators that span the realm of biological organization from genetic markers to entire ecosystems and are broadly applicable across geographic regions while integrating stressor types. We briefly review recent developments in ecologic indicators and emphasize the need for improvements in understanding of stress–response relationships, contributions of multiple stressors, assessments over different spatial and temporal scales, and reference conditions. We provide two examples of ecologic indicators that can improve our understanding of these inherent problems: *a*) the use of photopigments as indicators of the interactive effects of nutrients and hydrology, and *b*) biological community approaches that use multiple taxa to detect effects on ecosystem structure and function. These indicators are essential to measure the condition of coastal resources, to diagnose stressors, to communicate change to the public, and ultimately to protect human health and the quality of the coastal environment.

More than half the world’s human population resides within 100 km of the coastline [National Oceanic and Atmospheric Administration (NOAA) 1998; Vitousek et al. 1997], with increases likely over the next two decades (Stegeman and Solow 2002). The coastal zone represents at least half the value of global ecologic services (Costanza et al. 1997), and in economic terms, is the single most important source of recreational and residential income worldwide as well as fisheries (Jackson JBC et al. 2001; Ray and McCormick-Ray 2004).

Human development of coastal watersheds has greatly accelerated environmental pressure on downstream estuarine and coastal ecosystems; yet, unfortunately, assessing detrimental changes in these systems is complex. The symptoms of degradation include deterioration of water quality, loss of habitat and biodiversity, beach closings, fishery declines, fish consumption advisories, and an overall decline in the livability in the coastal zone [Boesch et al. 2001; Elofson et al. 2003; Hobbie 2000; Nixon 1995; National Research Council (NRC) 2000; Rabalais et al. 1996; Richardson 1997]. Coastal systems are hydrologically complex and are among the most susceptible to direct disturbance through global climate change. For instance, sea levels have been rising over the past century and even greater rises are predicted over the next 50 years (Jackson RB et al. 2001; Pilkey and Cooper 2004). These changes will continue to affect coastlines and will dramatically increase salt-water intrusion into freshwater coastal aquifers as well as the displacement of coastal agriculture (Jackson RB et al. 2001, McCarthy et al. 2001). Climate change is also believed to have increased events of heavy precipitation and flooding, which recently have become more common. These events increase the flushing of nutrients and toxic chemicals into coastal regions (McCarthy et al. 2001). In addition, many estuaries are highly urbanized and reference conditions are difficult or impossible to specify because of large changes in habitat, water diversion, and the introduction of exotic species (Nichols et al. 1986).

Not only are estuarine systems complex, but many stressors are also difficult to control. For example, non-point source nutrient pollution from coastal watersheds is a major problem, that has affected more than 60% of coastal rivers and bays (Howarth et al. 2000). Increases in nitrogen and phosphorus coming into coastal ecosystems have led to disruptions of basic ecologic functions [e.g., rising frequency and proliferation of harmful algal blooms (HABs) and increasing oxygen depletion (hypoxia)] with major damage to coastal fisheries and biodiversity (Jackson JBC et al. 2001; NRC 2000; Sundareshwar et al. 2003). In addition, nonpoint sources of toxic substances (e.g., agricultural chemicals and urban runoff) have impaired habitat quality for aquatic life and the human use of numerous coastal watersheds (Detenbeck et al. 1999; Kuivila and Foe 1995).

It is increasingly evident that ecosystem and human health are intricately linked (Stegeman and Solow 2002). For example, HABs can cause diseases in humans that result from consumption of contaminated seafood or inhalation of toxins entrapped in sea spray. Moreover, the distribution and frequency of HAB events have increased along U.S. coastlines over the last 30 years (Van Dohla 2000). People are also exposed to waterborne diseases through recreational contact, and the incidence of these diseases is increasing worldwide, as is the cost to the economy of frequent beach closures (Harvell et al. 1999). The principal agents of these diseases are bacteria, viruses, and protists. In the case of bacteria, this includes both native marine organisms (e.g., *Vibrio* species) and human or animal-derived pathogens from sewage and runoff (Rose et al. 2003). The importance of these connections between coastal conditions and human health was realized by the National Institute of Environmental Health Sciences (Research Triangle Park, NC) and National Science Foundation (Washington, DC), and they subsequently established their Centers for Oceans and Human Health.

Given the importance of coastal systems and the increasing pressure on them, quantitative measures of coastal ecologic conditions are absolutely essential for detection of change as well as for the design of control measures and restoration activities. Many approaches to comprehensive assessment of condition are cost prohibitive; thus, there has been a tendency to use broadly applicable indicators such as water clarity, nutrient or contaminant loads and levels, and various biodiversity measures such as species richness as metrics (NRC 2000; Whittier et al. 2002). Yet measurements of environmental condition are becoming more sophisticated and more applicable across space, time, and biological organization (Cottingham 2002). These new indicators include such metrics as diagnostic photopigments of algal functional groups to assess eutrophication (Paerl et al. 2002, 2003); biochemical and genetic indicators of toxicant exposure and stress (Anderson et al. 1994; Huggett et al. 1992; McCarthy and Shugart 1990); molecular techniques to assess human fecal bacterial distribution (Field et al. 2003); isotopic techniques to evaluate nutrient enrichment (Page 1995); indices of biological integrity or other biological community responses (Karr 1981; Simon 2003); ecosystem and population modeling approaches (Gentile et al. 2001); landscape metrics (DeAngelis et al. 1998; Whittier et al. 2002); and remote sensing techniques to detect large-scale land use impacts and change (Guerschman et al. 2003; Wolter and White 2002). While these indicators represent impressive advancements in both science and technology, there are limitations on their widespread and integrated use (NRC 2000).

Our primary goal in this review is to identify four critical areas in which scientific advancements are needed before improvements can be made in indicator development of coastal regions. In addition, we provide examples of two promising approaches to improvements in indicators. Defining the limitations of previous approaches and developing new approaches was a major goal of the U.S. Environmental Protection Agency (U.S. EPA) Science to Achieve Results Program (STAR) in establishing the Estuarine and Great Lakes (EaGLe) Research Program. The EaGLe investigators (Atlantic, Pacific, Gulf of Mexico, and the Great Lakes coastal areas) realize there is increasing urgency to develop indicators capable of detecting and diagnosing environmental conditions over space and time at cellular, organism, habitat, ecosystem, and regional levels. As the explosion of technical and conceptual advances in various disciplines ranging from molecular biology to ecosystems ecology and from remote sensing to bioinformatics continues to provide new and better tools, the goals of the EaGLe program and the science surrounding them are works in progress.

## Limitations of Current Coastal Indicators

**Figure 1:**
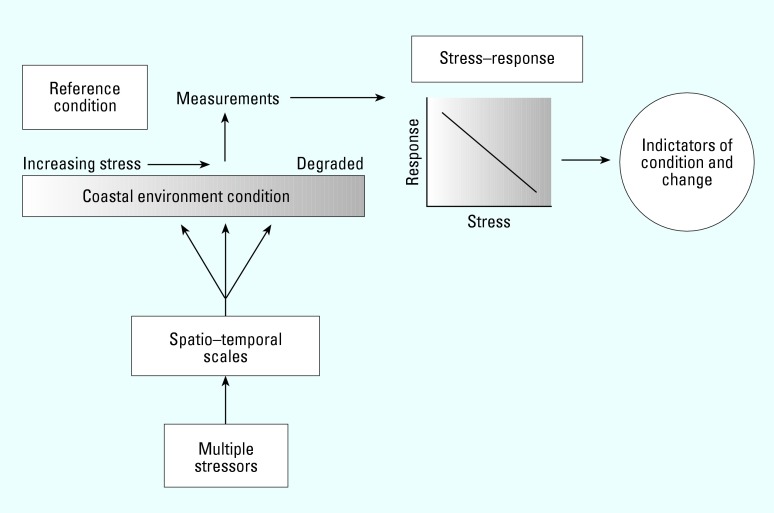
Conceptual diagram of critical elements in indicator development and the ultimate identification of indicators of condition and change. The sequence reflects multiple stressors that are distributed over different spatio–temporal scales within the coastal environment. The white rectangular boxes represent major limitations in the development of indicators as discussed in this review.

Most indicators were designed to provide specific information on local conditions such as water clarity or eutrophication or to provide broad-based snapshots of regional-scale water quality and habitat condition as exemplified by the total maximum daily load or Environmental Monitoring and Assessment Program (EMAP) (NRC 1994; U.S. EPA 1999). From these studies we know that large areas of the U.S. coastal zones are impaired (Bricker et al. 1999; Environment Canada and U.S. EPA 2003; U.S. EPA 2001), and many of the specific stressors within coastal watersheds that contribute to impairments have been identified (U.S. EPA 1999). Traditionally, the focus has been on indicators associated with water quality and toxic substances; however, there are major concerns on many other stressors, including habitat and landscape change, exotic species, global climate change, and pathogens. We focus on some of the limitations of current indicators that must be overcome for future advancements to be made on broad aspects of this problem in the coastal region (Figure 1):

### Stress with response.

Most current indicators of coastal condition are not linked with specific stressors; hence, it is unclear what causes the change in the indicator and, therefore, what management solutions should be implemented to affect an improvement.

### Multiple stressors.

Stressors in coastal ecosystems are diverse and originate from both anthropogenic and natural perturbations. Most current indicators are incapable of providing diagnostic information and are unable to discern the relative contribution of the various stressors to the observed changes in the indicators.

### Space and time.

Sources of stress to coastal environments operate over a range of spatial scales (e.g., square meters to entire landscapes) and time (seconds to decades). Current indicators are typically not explicit in how they relate condition with stressors over these different scales.

### Reference conditions.

The interpretation of the condition or change of an indicator is based on a comparison to reference conditions or benchmarks. Frequently, these reference conditions are not quantitatively defined; thus condition or meaning of a change in indicators is subject to considerable interpretation and debate.

## Linking Stress with Response

From a management perspective, among the greatest limitations of many indicators of coastal condition is the lack of a link with the cause for change (Suter et al. 2002). In the development of indicators, distinguishing between measurements of disturbance or stress and measurements of ecosystem response is imperative. The terminology in this area of the literature varies considerably, but recent reviews (NRC 2000; U.S. EPA 2003) provide clarification.

In developing stress–response relationships, natural experiments or surveys across a disturbance gradient from relatively pristine to highly disturbed areas are used most often (Karr and Chu 1999). In these situations, it is often difficult to control the stress because multiple factors often vary across any environmental gradient. Thus, inherent limitations in the scope of inference result and must be explicitly recognized. Understanding the response variable is also dependent on the strength of conceptual models used to describe the factors structuring the ecosystem, and on the extent to which anthropogenic disturbances influence that ecosystem. Obviously, detection of a response to a stressor is best accomplished by experiments in which the stressor can be manipulated in frequency, intensity, and duration. Unfortunately, these experiments often lack realism, as they are typically limited to laboratory situations or small-scale field-based mesocosms. However, a combination of a gradient design with field and laboratory experiments can be a powerful approach for the initial phases of indicator development, but as the indicator is applied at larger spatial scales or in uncharacterized sites, additional approaches are needed. Multivariate statistical approaches, novel modeling approaches, and techniques to aggregate indicators according to their use in management may all be valuable approaches. For example, these may permit more realistic diagnosis of stressors as complex as nutrient inputs, pathogen effects, and toxicant bioaccumulation.

A number of existing approaches are available to couple toxicant stress with response. For example, evaluation of the effects of toxic substances on ecosystems can involve multiple approaches: *a*) comparison of a toxic response or specific dose level of a contaminant to a water quality standard that has been linked to biological effects; *b*) assessment of environmental effects of multiple toxic substances by using well-characterized individual contaminant exposures; or *c*) the coupling of physiologic and genetic indicators with environmental chemistry and ecologic responses at multiple spatial and temporal scales. However, these too have their limitations. The first approach is limited when multiple contaminants and multiple stressor types are present. The second approach is primarily limited because often no direct integration of the toxic response and exposure occurs. The result is data correlation, which is also limited to the spatial and temporal scopes of the immediate investigation. The third approach considers multiple stressors and permits direct integration and scaling, but significant challenges remain to fully develop indicators for a range of habitats and model organisms. There are also combinations of these approaches. A simple index of sediment contamination, the Sediment Quality Triad, combines the first two. It provides a framework for analyzing benthic community data, analytical chemistry, and toxicity test data to assess whether a site is affected by toxicants, and is widely used throughout the nation (Long 2000; MacDonald and Ingersoll 2002). Yet, the action levels derived for specific contaminants are often unknown and benthic data are highly variable. Moreover, there is often a lack of specific reference conditions that precludes clear interpretation. The triad approach is also based on acute lethality, whereas sublethal effects can also be very important.

In summary, there is a need for controlled experiments in laboratory and field settings for those stressors (e.g., toxicants, nutrients, turbidity) amenable to manipulation. For larger-scale stressors such as introduction of exotic species, climate change, habitat loss, or landscape change such as fragmentation that are not easily amenable to manipulation, field experimental designs that test responses over gradients of stressor levels are among the options for linking stress with response (Danz et al., in press). Coupling the approaches of laboratory and field methods are essential for future development of appropriate response indicators.

## Multiple Stressors of Environmental Condition

Stress on coastal ecosystems is usually a combined effect of natural and anthropogenic disturbances. Natural disturbances in the U.S. coastal zones include water-level fluctuations from droughts and floods, wind events such as hurricanes, natural soil/sediment deposition, insect infestations, and forest fires. These natural disturbances vary in intensity both spatially and temporally. Major anthropogenic disturbances to the watershed of coastal ecosystems include permanent land cover conversions of native vegetation to agricultural, residential, and industrial areas; and temporary conversions of land due to forestry. These conversions result in concomitant disturbance to coastal ecosystems, including *a*) landscape effects of fragmentation, *b*) increased surface water runoff, *c*) increased nutrient and sediment input, *d*) increased pesticide and other chemical inputs, *e*) increased water temperature, and *f* ) greater human disturbance from recreational use, increased fish and shellfish extraction, and noise. Climate change and the resulting change in weather patterns is a combination of both natural, stochastic events, and human-induced warming which affects vegetation, water levels, and virtually all types of disturbance. In arid regions, water diversion and water use patterns also result in landscape and ecosystem-level alterations (Bennett and Moyle 1996). Detecting the effects of both individual disturbances and the simultaneous influences of natural and anthropogenic disturbances in coastal ecosystems is a challenging and complex task.

The relative effects of anthropogenic disturbance must be distinguished from the ranges of variation in natural disturbance regimes, but because of the large size and variability of coastal ecosystems, manipulative experiments to untangle the complexities of the varying disturbance regimes are difficult except on a relatively small scale. Combining specific indicators with modeling efforts can clarify and distinguish anthropogenic from natural stress in individual ecosystems and regions (DeAngelis et al. 1998; Gentile et al. 2001). However, the general trend has moved away from using direct diagnostic measures of stressors to using integrated indicators of ecosystem structure and function (NRC 2000). Yet, we know this approach may be inadequate for many applications because stressors vary among regions, implying that indicators are needed with diagnostic and prognostic capabilities. Indicators, therefore, could be grouped to fit regional needs related not only to assessing condition but also to developing appropriate management responses.

Characterizing the effects of multiple stressors on any ecosystem is among the most challenging tasks facing scientists today because multiple stressors can have synergistic, additive, or antagonistic effects on biological responses. Disentangling the various effects of multiple stressors will likely require a combination of controlled laboratory experiments, large-scale studies over multidimensional gradients of stress, and insightful modeling of ecosystem responses and change.

## Spatial and Temporal Explicitness

Ecologic indicators are constructed or selected to assess the condition of ecosystems and to detect environmental change related to human disturbance. Condition is often assessed by documenting the state or rate of ecologic processes such as productivity, respiration, or the structuring of biological communities. Indicators may do this by either measuring those processes directly (such as primary productivity) or inferring process from pattern (such as indices of biotic integrity (IBIs) as descriptors of community structure). Ecologic processes operate over a range of spatial and temporal scales, and the resulting patterns are expressed over varied scales. Hence, the relevant scale of each indicator must be specified to relate pattern to process in the appropriate conceptual model. Levin (1992) stated “the concepts of scale and pattern are ineluctably intertwined. The description of pattern is the description of variation, and the quantification of variation requires the determination of scales.”

Many studies have sought to quantify spatio–temporal patterns across a range of scales. Unfortunately, few have determined whether patterns are consistent across scales or whether related phenomena cross scales (Caldow and Racey 2000). In addition, because of technical, logistical, and financial reasons, most ecologic studies have focused on small systems (e.g., site or plot level) and short periods of time, which in turn has limited the development over large spatial scales (Innes 1998). Alternatives such as top-down approaches do exist. Here, large-scale processes form the basis of inferring process from pattern and are being applied in regional classification schemes (Hawkins et al. 2000).

The need for scale explicitness is complicated by multiple stressors arising from human and natural disturbances. Most ecologic indicators are related to multiple stressors and scales. For example, in a study of littoral macroinvertebrate communities (Johnson and Goedkoop 2002), 23% of the variance in taxonomic composition was associated with habitat factors, but greater spatial scales (riparian, catchment, ecoregion classification) accounted for 24% of the variance. If indicators are to be used effectively in management, it is necessary that we know the relevant scale(s), so that the scale of management actions matches the scale of the phenomena being measured (Hobbs 1998). Experimental approaches that allow for the partitioning of the variance among different stress components and over a hierarchy of spatial scales will be critical in the development of new ecologic indicators.

## Reference Conditions

To interpret any set of indicators, one must compare results of monitoring to standards or benchmarks. One of the preferred benchmarks is a reference site or condition. The use of reference sites has become increasingly common as ecologists and managers search for reasonable and scientifically based methods to measure and describe the inherent variability in natural aquatic systems (Kentula et al. 1992; Rheinhardt et al. 1999). As there are likely few places on earth unaffected by anthropogenic disturbances, true reference areas remain elusive. For example, even coastal regions of Greenland or Antarctica have been affected by atmospheric chemical inputs and climate change. Alternatively, even in coastal regions with long histories of human occupation and, hence, anthropogenic disturbances, reference sites can be established using specific estuaries, watersheds, or lotic systems entering the coastal zone. These may represent the best attainable environmental conditions for a specific geographic setting, a historic representation using paleolimnologic data, a simulated reference condition, or a situation where conditions fall within the range of natural variability for the system.

Determining reference condition of a system is highly dependent on the indicators used and the locations where samples were gathered. Benthic indicators will provide different results than fish indicators. Similarly, indicators will be different in large, ephemerally stratified systems (e.g., Chesapeake Bay, Maryland–Virginia; Pamlico Sound, North Carolina; Mobile Bay, Alabama; San Francisco Bay, California; or Green Bay, Wisconsin) compared with smaller, well-flushed systems. For example, phytoplankton growth responses to nutrient enrichment will not be as profound as those for benthic microalgae in well-flushed systems. Here, benthic microalgae may be more sensitive and meaningful indicators of ecosystem response to nutrient enrichment. Indicators of community structure (i.e., diversity indices, keystone species) may gauge ecosystem conditions quite distinct from indicators of function (e.g., primary and secondary production, respiration, and nutrient cycling). IBI, habitat suitability indices, and chemical monitoring are specific examples of indicators that in combination can assess structure, physical–chemical quality, and biological measures of reference condition.

Historical information from a site, such as survey data, paleolimnologic studies, and habitat reconstruction, can be extremely helpful for determining reference conditions. Finding such information, however, can also be very difficult and most historical information is not quantitative such as for urban estuaries (Nichols et al. 1986). Hughes (1995) summarized the basic characteristics necessary for a suitable reference condition including reasonableness and political acceptability, sufficient number of reference sites within the area of interest, and suitable data on natural conditions of the site.

## Examples of New Indicators

Environmental indicators can have an enormous number of possible end points, reflecting the breadth and diversity of the scientific underpinnings in biology, chemistry, and physics (McKenzie et al. 1992; Noss 1990; NRC 2000; O’Neill et al. 1988). For example, biological indicators span the realm of biological organization from genetic markers to entire ecosystems. Chemical indicators reflect a variety of spatial or temporal scales ranging from oxygen demand for a specific point source to global carbon dioxide distributions in the atmosphere. Physical indicators can include elevational, morphologic, transport, circulation, exchange and stratification processes with all their attendant ramifications for ecosystem structure and function. Because of the massive amounts of information that can be gathered across levels of physical, chemical, and biological organization and across spatial or temporal scales (Dixit et al. 1992; Karr and Chu 1999), the challenge to integrate data across levels of organization in space and time is daunting.

Current programmatic and academic funding scenarios exacerbate our lack of integration. Most funding is limited by amount and duration. These monetary and time deficiencies have been recognized by academic funding sources, such as NSF (e.g., Long Term Ecological Research, Biocomplexity, Global Ocean Flux, Biotechnology, and other centers) and U.S. EPA (EaGLe). As such programs mature, advances in integrating across a variety of trophic levels as well as organizational or spatial and temporal scales will likely occur.

We provide two brief examples of new types of indicators; one that links productivity (function) and hydrology and another linking community (structural) patterns. We believe these new types of indicators will substantially improve our ability to measure and understand the complexity, response, and condition of coastal systems. For example, analyses of photopigments provide a means to explicitly link nutrient and hydrologic stressors with specific phytoplankton groups and over explicit spatial scales when combined with remote sensing information. If data are gathered systematically over time, then temporal changes can also be linked with specific stress events (e.g., hurricanes). In the second example, multitaxa types of approaches provide a wide range of possibilities for improving our knowledge of stress–response relationships, the identification of multiple stressor effects, spatial and temporal explicitness, and the identification of suitable reference conditions. For example, Luoma et al. (2001) provide eight case studies that specify cause and effect linkages using community analyses of macroinvertebrates to decipher the effects of multiple stressors. Paleolimnologic data derived from sediment and water column sampling of diatom communities are among the most powerful techniques for identifying suitable reference conditions within aquatic systems (Dixit et al. 1992).

## Photopigments as Integrators of Estuarine Nutrients and Hydrology

Nitrogen availability most frequently controls microalgal and higher plant primary production in estuarine and coastal waters (Nixon 1995; Ryther and Dunstan 1971). Loading rates of this nutrient directly reflects human population density and activity in coastal water- and airsheds (Peierls et al. 1991). Excessive nitrogen loading is a key causative agent for accelerating primary production or eutrophication (Nixon 1995; Paerl 1997). Symptoms include phytoplankton blooms, which may accumulate as ungrazed organic matter in the sediments, providing the “fuel” for oxygen consumption and depletion in bottom waters and sediments. This chain of events is particularly problematic in salinity- or temperature-stratified waters, where oxygen may not be easily replenished from the atmosphere. Hypoxic conditions alter nutrient cycling and promote fish disease and mortality (Paerl et al. 1998).

Suspended microalgae or phytoplankton account for the bulk of estuarine and coastal primary production in many estuarine and coastal ecosystems. Their composition and activity are key in determining fertility, eutrophication, and water quality. Water discharge controls transport of phytoplankton through these systems and plays an interactive role with nutrient supply to control phytoplankton growth, competition, succession, and community composition. For example, high rates of freshwater discharge reduce the salinity and residence time. These conditions favor fast-growing oligohaline phytoplankton, such as chlorophytes (green algae). In contrast, low-discharge conditions promote long water residence, high salinity conditions, which favor slower growing halophylic taxa such as dinoflagellates and certain cyanobacteria. Phytoplankton community composition affects the structure and function of estuarine food webs, nutrient cycling, habitat condition, fishery resources, and overall ecosystem condition (Paerl et al. 2002, 2003).

Chlorophyll *a* has been used for many years as a sensitive indicator of phytoplankton biomass. However, because virtually all phytoplankton contain this pigment, it alone cannot be used to determine community composition. Using additional diagnostic chlorophyll and carotenoid photopigments as indicators of major phytoplankton functional groups (i.e., diatoms, dinoflagellates, chlorophytes, cyanobacteria, cryptomonads), we can examine the interactive effects of nutrient and hydrologically driven changes of phytoplankton community composition and activity. HPLC, coupled to photodiode array spectrophotometry is used to determine phytoplankton group composition based on the diagnostic photopigments. Photopigment markers include chlorophyll *b* and lutein (chlorophytes), zeaxanthin, myxoxanthophyll, and echinenone (cyanobacteria), fucoxanthin (diatoms), peridinin (dinoflagellates), and alloxanthin (cryptomonads). A statistical procedure, ChemTax (Mackey et al. 1996), partitions chlorophyll *a* (i.e., total microalgal biomass) into the major algal groups to determine the relative and absolute contributions of each group.

**Figure 2:**
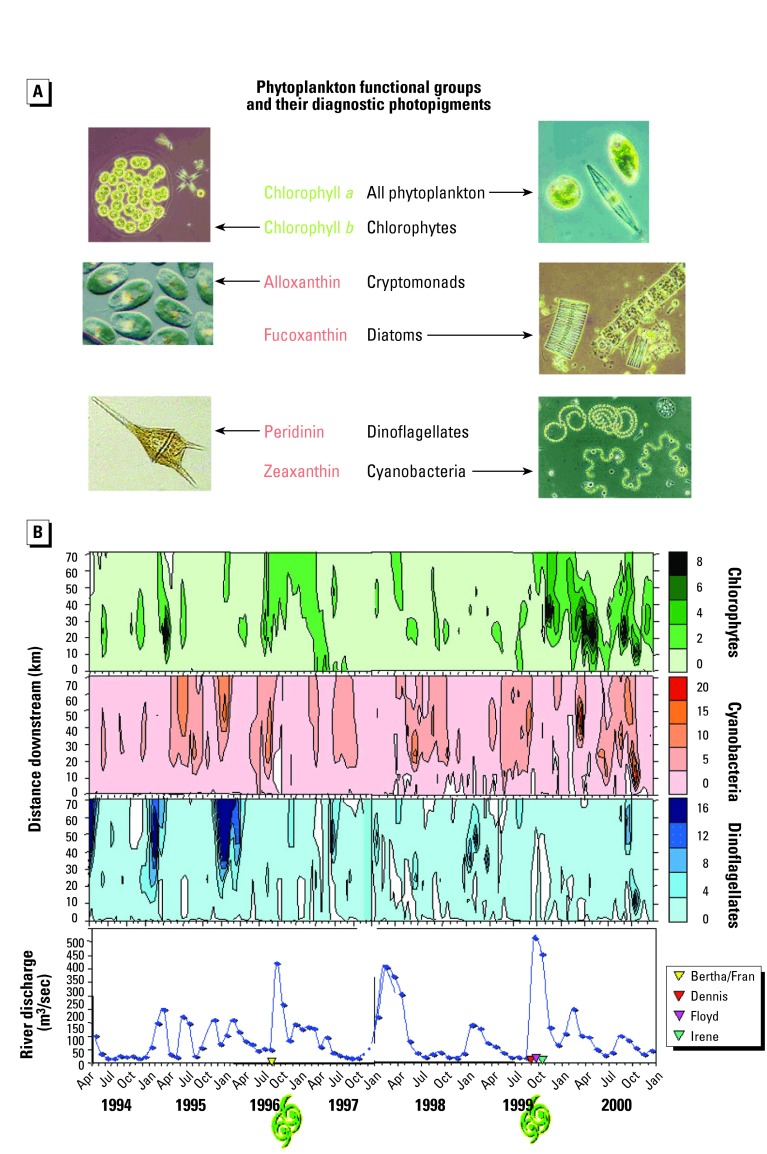
(*A*) Chlorophyll and carotenoid photopigments are diagnostic of major estuarine phytoplankton groups. Since chlorophyll a is present in each of the groups, it is used to quantify total phytoplankton biomass. The individual carotenoids and some chlorophylls (e.g., chlorophyll b) can be used to distinguish and quantify individual phytoplankton functional groups, using the matrix factorization program ChemTax Mackey 1996). (*B*) Distributions, in time and space, in the Neuse River estuary of chlorophyll *a*, contributed by several phytoplankton functional groups dominating primary production between 1994 and 2000. Groups shown here are chlorophytes, cyanobacteria, and dinoflagellates. Values were derived using ChemTax for surface water at midestuarine mesohaline locations sampled by the MODMON program (Neuse River Estuary and Monitoring 2001). Biweekly data were temporally extrapolated along the axis of the estuary, from its freshwater head at New Bern, North Carolina (0 km), to a downstream mesohaline location near the entrance to Pamlico Sound. Freshwater discharge entering the estuary is also shown. The dates are shown of landfall of the four major hurricanes that have significantly affected flow and nutrient enrichment since mid-1996.

Examples from ongoing studies in the Neuse River estuary in North Carolina and Pamlico Sound (1994–present) show that these systems have experienced the combined stresses of anthropogenic nutrient enrichment, droughts (reduced flushing combined with minimal nutrient inputs), and since 1996, elevated hurricane activity (high flushing accompanied by elevated nutrient inputs) (Figure 2). Seasonal and hurricane-induced variations in river discharge, and the resulting changes in flushing rates, and hence, estuarine residence times, have differentially affected phytoplankton taxonomic groups as a function of their contrasting growth characteristics. For example, the relative contribution of chlorophytes, cryptophytes, and diatoms to the total chlorophyll *a* pool was strongly controlled by periods of elevated river flow (Figure 2). These effects are due to the efficient growth rates and enhanced nutrient uptake rates of these groups. Cyanobacteria, on the other hand, showed greater relative biomass when flushing was minimal (i.e., longer residence times) during the summer.

Further evidence that hydrologic changes have altered phytoplankton community structure is provided by the observed historical trends in dinoflagellate and chlorophyte abundance. Both decreases in the occurrence of winter-spring dinoflagellate blooms and increases in the abundance of chlorophytes coincided with the increased frequency and magnitude of tropical storms and hurricanes since 1996. The relatively slow growth rates of dinoflagellates may have led to their reduced abundance during these high river discharge events. These changes in the phytoplankton community have been linked to altered trophodynamics and nutrient cycling, which subsequently affects fishery habitats and yields.

Diagnostic photopigment analyses are able to detect significant changes in phytoplankton community composition over a broad range of time scales (< 24 hr to decades) and as such are well suited for monitoring programs designed to assess short- and long-term trends in water quality in response to: hydrographic features (circulation, upwelling); nutrient enrichment; climatic; and hydrologic perturbations (floods, droughts). In addition “top down” effects of grazing have been examined using an HPLC-based technique. Finally, these analyses have proven useful as a means of ground-truthing and calibrating remotely sensed estimates of phytoplankton bloom events (Harding et al. 1999; Millie et al. 1997). This coupling of indicator technology with remote sensing enables “scaling up,” namely, mapping the spatial distributions of phytoplankton groups over large geographic areas not amenable to routine field sampling, evaluating the effectiveness of nutrient management strategies, use as an early warning system for blooms of nuisance or toxic species (Millie et al. 1997), and as a sensitive bioindicator of overall water quality conditions (Pinckney et al. 2001; Paerl et al. 2003).

## Biological Community Responses as Condition and Change Indicators

Plant and animal community structure and function have been extensively measured to describe the condition of both aquatic and terrestrial systems. The major challenge is how to scale and aggregate the responses of species populations at the site level to reflect conditions of the biological community level for specific taxa or to provide assessments of large scale patterns, such as IBI (Karr 1981), biological species profiles (Simon 2003), multitaxa indices (O’Connor et al. 2000), or indices of environmental integrity (Paul 2003). These approaches hold tremendous potential for assessments of environmental conditions over large landscape or regional areas, as well as for detection of temporal change. However, these indicators will also require considerable development in the areas of *a*) providing linkages with single and multiple stressors; *b*) exploration of analytical techniques to integrate and synthesize multiple biological signals from species or functional groups within the biological community; *c*) parsing these multivariate responses among stressors and over varying spatial scales; and *d*) providing explicit spatial or temporal scales for the indicators which are consistent with the scales of management actions (Niemi and McDonald, in press).

The strength of the community approach lies in the differential sensitivity of individual species, functional groups (e.g., guilds), or trophic levels to different stressors. Each of these levels can respond differently to stress. For example, O’Connor et al. (2000) found a correlation among many taxa (diatoms, benthos, zooplankton, fish, birds) to the gross condition of lakes, but fish provided the best measure of condition in the near-shore environment. In an experiment on the pesticide effects of mosquito control agents in wetlands, of the zooplankton, aquatic insect, and bird communities studied, only the aquatic insects exhibited a response to treatment (Hershey et al. 1998; Niemi et al. 1999). In this case, aquatic insects were the best indicator of pesticide effects. Niemi and McDonald (in press) provide many examples of responses by different taxa to diverse stressors.

The unique aspect of the community approach is the ability to sample a wide variety of taxa; each of which has a unique life history capable of being disrupted by stress at various scales. All coastal regions are represented by thousands of species including taxa such as bacteria, plankton, macroinvertebrates, fish, vascular and nonvascular plants, amphibians, and birds. Many associations between these taxa and stress exist. For example, diatoms are particularly sensitive to nutrients (Dixit et al. 1992), whereas benthic invertebrates are responsive to sediment contamination in both lakes and estuaries (Bailey et al. 1995, Rakocinski et al. 1997). Fish communities are sensitive to human development (Brazner 1997) and exotic species (Rahel 2000). Wetland vegetation is directly affected by hydrologic modifications such as dikes and road building (Herdendorf 1992). Amphibians are sensitive to water quality in wetlands (Kutka and Bachman 1990). Bird populations are affected by landscape-level habitat change and fragmentation (Robinson et al. 1995). Moreover, many of these taxa have well-established sampling methods, and some have long-term nationwide monitoring programs that are currently still in use (Likens 1989; Robbins et al. 1989). The combination of species- or taxa-specific responses by plant and animal communities to stressors and the availability of extant monitoring programs allows for the partitioning of multiple stress–response relationships.

Probability-based and standardized sampling of communities within specific sites but over large landscapes have proven useful for regional-scale assessments of environmental conditions (Olsen et al. 1999). For example, these approaches have been used for the identification of imperiled systems (Stein et al. 2000); development of biological indicators for the Mid-Atlantic Highlands (Herlihy et al. 1998); and establishing the condition of streams and estuaries through U.S. EPA’s EMAP (U.S. EPA 2002). One important aspect of these large-scale approaches is the development of indicators that can identify areas that have the most severe problems and greatest need of management attention, action, and potentially restoration. Integration of these types of data will be challenging and will require multivariate, integrative approaches of multitaxa biological communities over large-scale landscapes and regions.

Promising new techniques to achieve integration of community measurements over multiple spatial, temporal, and biological scales are evolving such as development of multimetric indices (e.g., Karr 1981; Paul 2003), and statistical techniques (Jongman et al. 1995). These techniques will require coupling with population and ecosystem-based models for aid in the interpretation of stressor risk and alternative management actions (DeAngelis et al. 1998; Gentile et al. 2001). Several large-scale programs have been initiated such as the EaGLe program reported here. For example, Danz et al. (in press) developed a stratified experimental design for the development of environmental indicators in the Great Lakes coastal region. This design was based on the compilation of more than 200 data layers on stress information for 762 coastal units and the identification of gradients of stress for several coastal ecosystem types. Six different taxa (amphibians, birds, diatoms, fish, macroinvertebrates, vegetation) were randomly sampled across these stress gradients to detect differential responses. This work is still in progress but identifies the tremendous potential of spatially explicit public databases in the future development of environmental indicators. With the exponential increase in technological and computational capabilities including molecular techniques, remote sensing technology, modeling and statistical sophistication, data management and storage, and internet communication, analysis of biological communities as indicator signals of environmental condition and change will rapidly advance.

## Conclusions

Coastal ecosystems have experienced tremendous stress from natural and anthropogenic influences for hundreds of years, and stress levels are projected to increase in the future. A new generation of ecologic indicators is needed to measure the condition, diagnose stressors, communicate condition to the public, assess potential future status, and evaluate management actions in our coastal regions. Among the many challenges, the development of these indicators will require improvements in our scientific understanding of stress–response relationships, relative contributions of multiple stressors, how stressors operate over different organizational and spatio–temporal scales, and how reference conditions are determined. Fortunately, there is an explosion of ideas and technology that can aid in the multidisciplinary advancement of indicators in our coastal waters.
